# Genetically predicted circulating linoleic acid levels and risk of osteoarthritis: a two-sample mendelian randomization study

**DOI:** 10.1186/s12891-024-08018-4

**Published:** 2024-11-13

**Authors:** Wen Yang, Wenwu Xiao, Hailong Liu

**Affiliations:** 1 Department of Rehabilitation Medicine, Affiliated Renhe Hospital of China Three Gorges University, Yichang, 443001 Hubei China; 2 Department of Rehabilitation Medicine, the First Affiliated Hospital of Sun Yat- sen University, Guangzhou, 510080 Guangdong China; 3 Department of Joint Surgery, the First Affiliated Hospital of Sun Yat-sen University, Guangzhou, 510080 Guangdong China

**Keywords:** Osteoarthritis, Linoleic acid, Mendelian randomization

## Abstract

**Objectives:**

This study aimed to provide insight into the effect of genetically predicted linoleic acid (LA) levels on osteoarthritis (OA).

**Methods:**

The LA dataset was obtained from the UK Biobank (UKBB) consortium and contained 114,999 samples. The OA discovery dataset was derived from MRC-IEU consortium and included 38,472 cases and 424,461 controls. The OA validation set was derived from a summary-level genome-wide association study (GWAS) and included 39,427 cases and 378,169 controls. Genetic variants strongly associated with LA (*p* < 5 × 10^− 8^) were extracted as instrumental variables (IVs). The inverse variance weighted (IVW) approach was adopted as the primary analysis method in this study. In addition, multiple sensitivity analysis methods were used to assess the reliability of our results.

**Results:**

The IVW approach showed that circulating LA levels were negatively associated with OA risk in the discovery set (odds ratio (OR) = 0.993, 95% confidence interval (95% CI): 0.988–0.998, *p* = 0.011). A consistent result was obtained in the validation set (OR = 0.904, 95%CI: 0.845–0.967, *p* = 0.003). These results were validated by sensitivity analysis.

**Conclusion:**

This study provides new evidence for the causal relationship between LA and OA, which provides new insights for the treatment of OA.

**Supplementary Information:**

The online version contains supplementary material available at 10.1186/s12891-024-08018-4.

## Introduction

Osteoarthritis (OA) is a joint disease that can cause acquired disability in adults and is prevalent in older women, most commonly in the hip and knee joints [1]. It is reported that approximately 7% of the world’s population is affected by OA, representing a significant burden to individuals and society [2]. The pathogenic factors of OA are complex, and age, gender, obesity, mechanical factors and genetic susceptibility are recognized risk factors for OA [3]. However, to date, there is no effective treatment other than joint replacement surgery.

Fatty acids (FAs) are the fat-soluble fraction of plants or animals and are the major components of lipids. FAs can be classified as saturated fatty acids (SFAs) or unsaturated fatty acids (UFAs) based on the presence or absence of double bonds. UFAs can be classified as monounsaturated fatty acids (MUFAs) and polyunsaturated fatty acids (PUFAs) according to the number of double bonds. Based on the position of the last double bond relative to the methyl group at the end of the molecule, PUFAs can be further classified into omega-6 and omega-3 PUFAs. A recent review summarizes the different roles of different types of fatty acids in OA [4]. Among these, omega-6 PUFAs are thought to play a pro-inflammatory role, while omega-3 PUFAs have an anti-inflammatory effect [5]. Linoleic acid (LA; 18:2n-6, octadecadienoic acid), a key fatty acid of the omega-6 family, is the most abundant PUFA in the human diet [6]. In the last 60 years, the average LA intake in Western countries has risen almost 10-fold (about 4–10% of total dietary calories). Since it cannot be synthesized in the human body, it needs to be obtained from external sources, such as vegetable oils and products made from vegetable oils. Previous studies have confirmed that high LA diets are unhealthy and promote the development of inflammation. However, several recent studies have shown that not only is there no significant positive correlation between LA intake and several inflammatory markers, but it also reduces the expression of inflammatory markers. This challenges the conventional wisdom that linoleic acid promotes the progression of the inflammatory response. However, the role of LA in OA is unclear. Several studies have suggested a potential OA-promoting role for LA. An in vitro study showed that LA may induce inflammatory mediators in chondrocytes, and thereby promote OA progression [7]. LA treatment of chondrocytes inhibited chondrocyte autophagy and promoted cartilage degeneration [8]. A recently published study showed that linoleic acid levels in the synovial fluid of patients with OA combined with obesity were significantly higher than those of patients with OA alone in mass spectrometry analysis [8]. Anne et al. also found elevated levels of LA in equine OA synovial fluid [9]. In contrast, a newly published exploratory metabolomic study revealed that LA levels were instead elevated after OA was reduced [10]. Another cohort study found that LA levels were negatively associated with hand OA severity [11]. These seemingly contradictory conclusions may be due to the limitations of observational or interventional studies, such as confounding factors, measurement error, and reverse causality. Therefore, there is an urgent need for a new approach to eliminate these confounding factors, thus helping us to explore the relationship between LA and OA more rigorously.

Mendelian randomization (MR) is a recently popular analytical method that inferred the potential causal impact of risk factors on disease by using a number of genetic variants as instrumental variables (IVs) [12, 13]. These genetic variants are randomly assigned at the time of conception, and thus MR analysis could circumvent potential confounders (such as environment or lifestyle) and reverse causality bias [14, 15].

Currently, MR analysis has been widely used to investigate risk factors associated with OA. However, no studies have investigated the causal relationship between LA and OA. In this study, we conducted a two-sample MR analysis to investigate the causal relationship between LA and OA using the most recent and largest sample size of summary-level data.

## Methods

### Study design and data sources

This research is based on data from recently published large-scale genome-wide association study on circulating LA levels and OA. We also selected the two most common types of OA, knee OA(KOA) and hip OA(HOA), for more precise subtype analysis. The basic information of all datasets is shown in Table 1.

**Table 1:** Basic information of genome-wide association studies (GWAS) datasets

Exposures	Consortium	Ancestry	Sample size	SNPs	F-statistic
LA	UK Biobank	European	114,999	57	118
**Outcomes**	**Data sources**	**Ancestry**	**Sample size**	**Cases**	**Control**
OA (discovery)	MRC-IEU	European	462,933	38,472	424,461
OA (validation)	UK Biobank, arcOGEN, UKHLS	European	417,596	39,427	378,169
KOA	UK Biobank, arcOGEN, UKHLS	European	403,124	24,955	378,169
HOA	UK Biobank, arcOGEN, UKHLS	European	393,873	15,704	378,169

**Fig. 1:**
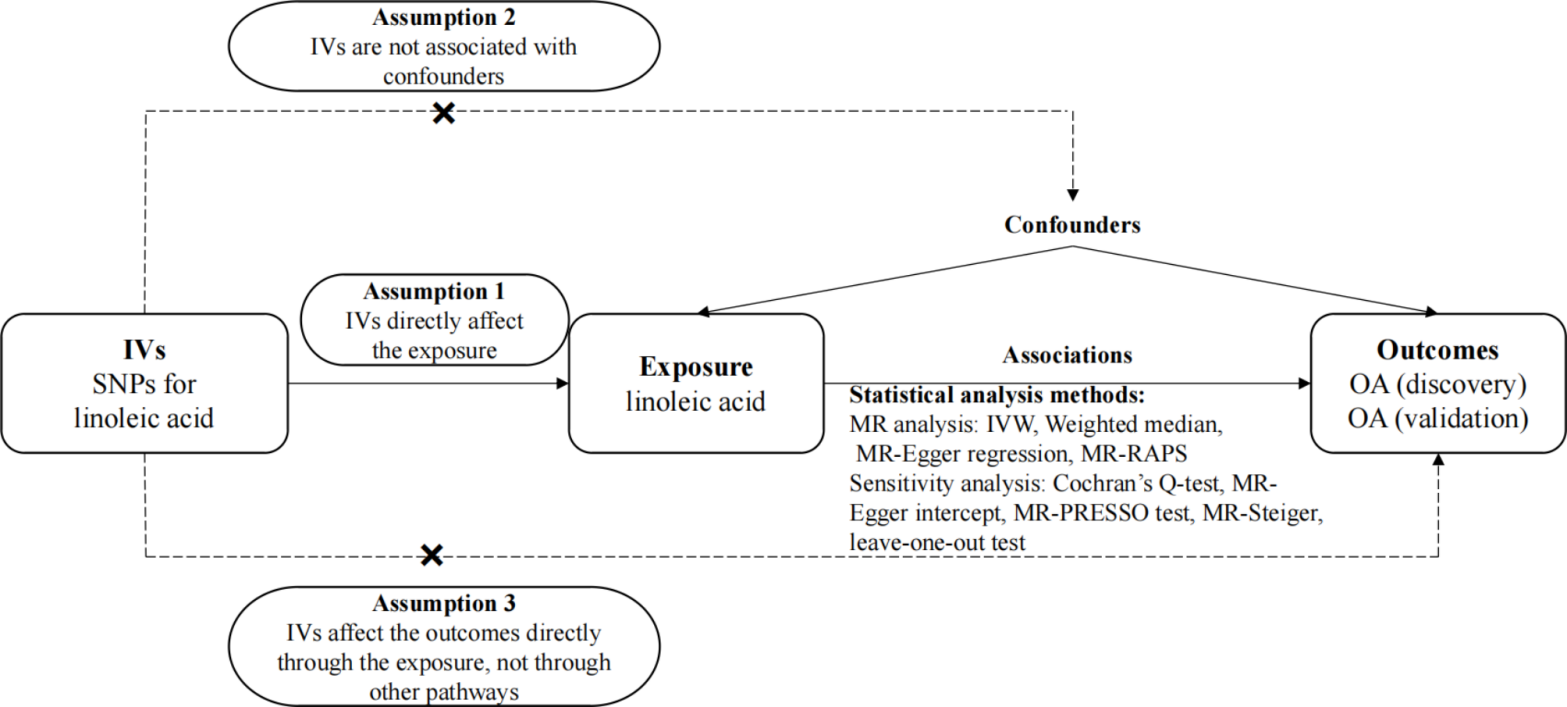
Flow chart of this study

Our MR analysis was conducted with the following three main assumptions. (1) IVs should be strongly correlated with exposure. (2) IVs should be independent of potential confounders. (3) IVs do not directly affect the outcome, but only through exposure. Ethical approval and informed consent have been received for the original studies, and therefore were not required for this research. The flowchart of our study is shown in Fig. 1.

### Selection of Instrumental Variables (IVs)

The IVs for LA were derived from UKBB and included 114,999 samples. We screened for single nucleotide polymorphism (SNPs) with genome-wide significance (*P* < 5 × 10^− 8^). Subsequently, to ensure the independence and validity of IVs and reduce the effect of strong linkage disequilibrium (LD), r2 < 0.001 and lumping distance > 10,000 kb were set as the thresholds of the lumping algorithm. Furthermore, we searched each SNP using the Phenotype Scanner database [16] and excluded IVs associated with potential confounders (e.g., obesity, type 2 diabetes, body mass index, hypertension, etc.) of OA [17, 18]. The F-statistic [19, 20] was used to assess the strength of the correlation between IVs and LA. F-statistic > 10 indicated a relatively strong effect of IVs, and IVs with F < 10 were excluded. The details of selected IVs for LA are shown in Supplemental Table S1.

### Outcome data sources

Summary-level GWAS data for OA were obtained from the MRC-IEU consortium, which included 462,933 samples (38,472 cases and 424,461 controls). The MAF threshold was set at 1% as the SNP inclusion criteria for OA. This was the largest sample size of GWAS data on OA publicly available, and we used it as a discovery set. Another summary-level data [21] on OA came from a UKBB study that integrated data from UKBB, arcOGEN, and UKHLS. This study contained 417,596 samples (39,427 cases and 378,169 controls) and had the same selection criteria as the discovery set. We used it as the validation set. GWAS data on the two subtypes KOA and HOA were also obtained from the UKBB, arcOGEN, and UKHLS database.

### Two-sample MR analysis

The inverse variance weighted (IVW) method was adopted as the primary method in this study, because it analyzed the Wald ratio of each IV on the outcome and was considered the most efficient [22, 23]. In addition, four other analysis methods were also used for repeated analysis, including the MR-Egger [24], weighted median (WM) [25], MR-robust adjusted profile score (MRRAPS) [26], and MR-pleiotropy residual sum and outliers (MR-PRESSO) [27]. MR-Egger analysis can evaluate causal effects and horizontal pleiotropy. When 50% of the SNPs are invalid, the WM method still allows reliable causal estimation. The MR-RAPS method takes into account the measurement error of the SNP exposure effect and is robust to system and specific pleiotropy. MR-PRESSO method was adopted to find and remove outliers (*p* < 0.05) and to calculate corrected causal effects. To validate the robustness of the results, we conducted several sensitivity analyses. Cochran’s Q statistic was used to assess the heterogeneity among SNPs [24, 28]. When the Q statistic of Cochrane was *p* < 0.05, the multiplicative random effects IVW method was used; otherwise, the fixed model should be used. The MR-Egger intercept test was used to the evaluate the horizontal pleiotropy [27]. MR Steiger filtering test was used to determine the direction of causal effects [29]. Furthermore, leave-one-out analysis was used to evaluate whether the causal effect was driven by a single SNP [24].

### GO, KEGG, PPI network analysis and hub genes screening

To further explore the underlying biological mechanisms, we performed some bioinformatics analyses. Gene Ontology (GO), which involved biological processes (BP), cellular components (CC) and molecular functions (MF), and Kyoto Encyclopedia of Genes and Genomes (KEGG) pathway enrichment analysis were conducted by using R package “clusterProfiler”, and the threshold values of p and q were set as 0.05. The results were visualized by R package “ggplot2”. STRING (version12.0, https://cn.string-db.org/) was used to construct the protein-protein interaction (PPI) network with a confidence score > 0.4. Cytoscape (version3.8.2) was used for visualization and the plug-in cytoHubba was used to calculate the ranking of mapping genes. We selected the top 10 genes of the MCC method.

### Statistical analysis

We manually removed potential confounding variables and used instrumental variables for causal analysis. The effects of LA on OA, KOA and HOA risk were represented by odds ratio (OR) and 95% confidence interval (95%CI). A two-sided *p*-value < 0.05 was considered statistically significant. All MR analyses were performed using the “TwoSampleMR” and “MR-PRESSO” packages in R software (version 4.1.3).

## Results

### Screening for IVs

A total of 33 SNPs associated with LA were finally extracted as IVs in the discovery set, while 40 SNPs were extracted in the validation set. In addition, a total of 40 LA-related SNPs were extracted as IVs in the KOA set, while 38 SNPs were extracted in the HOA set. Detailed information on the IVs is shown in Supplemental Table S1. The F-statistics of all screened SNPs were greater than 10, indicating that there were no weak IVs.

### Main MR analysis

**Fig. 2:**
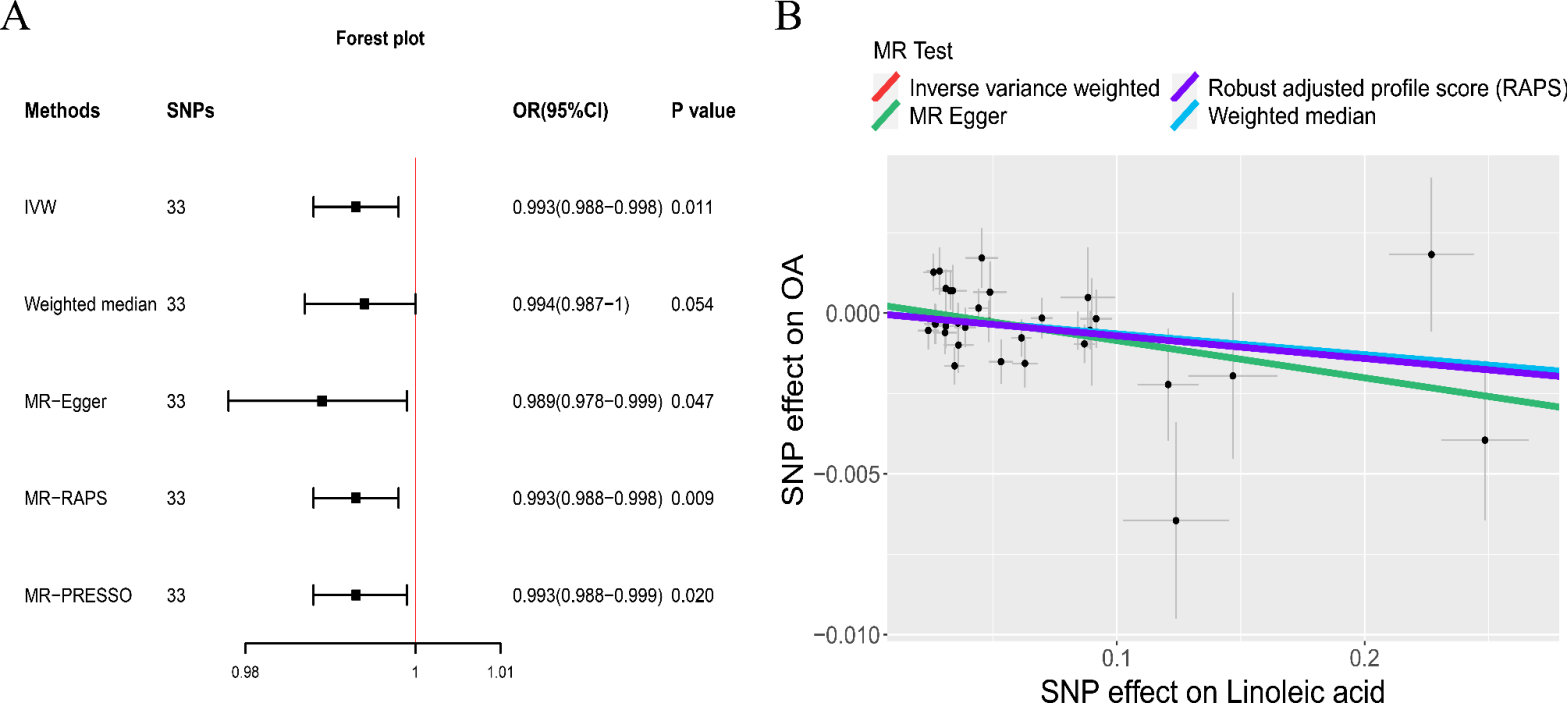
The causal relationship between linoleic acid and osteoarthritis (discovery). Forest plot (**a**) Scatter plot (**b**)

As shown in Fig. 2, the IVW method indicated that Genetically predicted higher LA levels were correlated with a significantly lower risk of OA. For 1 standard deviation (SD) increment of LA, the OR and 95% CI for OA were OR = 0.993 (95% CI: 0.988–0.998, *p* = 0.011). MR Egger, MR-RAPS and MR-PRESSO methods derived the same results as the IVW method. The MR-Egger intercept method revealed no horizontal pleiotropy (Table 2). MR Steiger test showed that all IVs were “TRUE”, which confirmed our assessment of the potential causal direction (Supplemental Table S2). In addition, the leave-one-out analysis showed that single SNP did not lead to causal bias, which further demonstrated the robustness of our results (Supplemental Figures S1).

**Table 2:** Heterogeneity and horizontal pleiotropy test of LA IVs on the risk of osteoarthritis

Exposure	Outcome	Heterogeneity test				Pleiotropy test	
		IVW		Egger		Egger- intercept	
		Q-statistics	*p* value	Q-statistics	*p* value	intercept	*p* value
LA	OA (discovery)	44.599	0.069	43.317	0.070	0.0003	0.345
	OA (validation)	62.494	0.010	61.738	0.009	0.0026	0.499
	KOA	57.531	0.028	57.512	0.022	0.0005	0.912
	HOA	40.090	0.335	39.024	0.335	0.0049	0.328

**Fig. 3:**
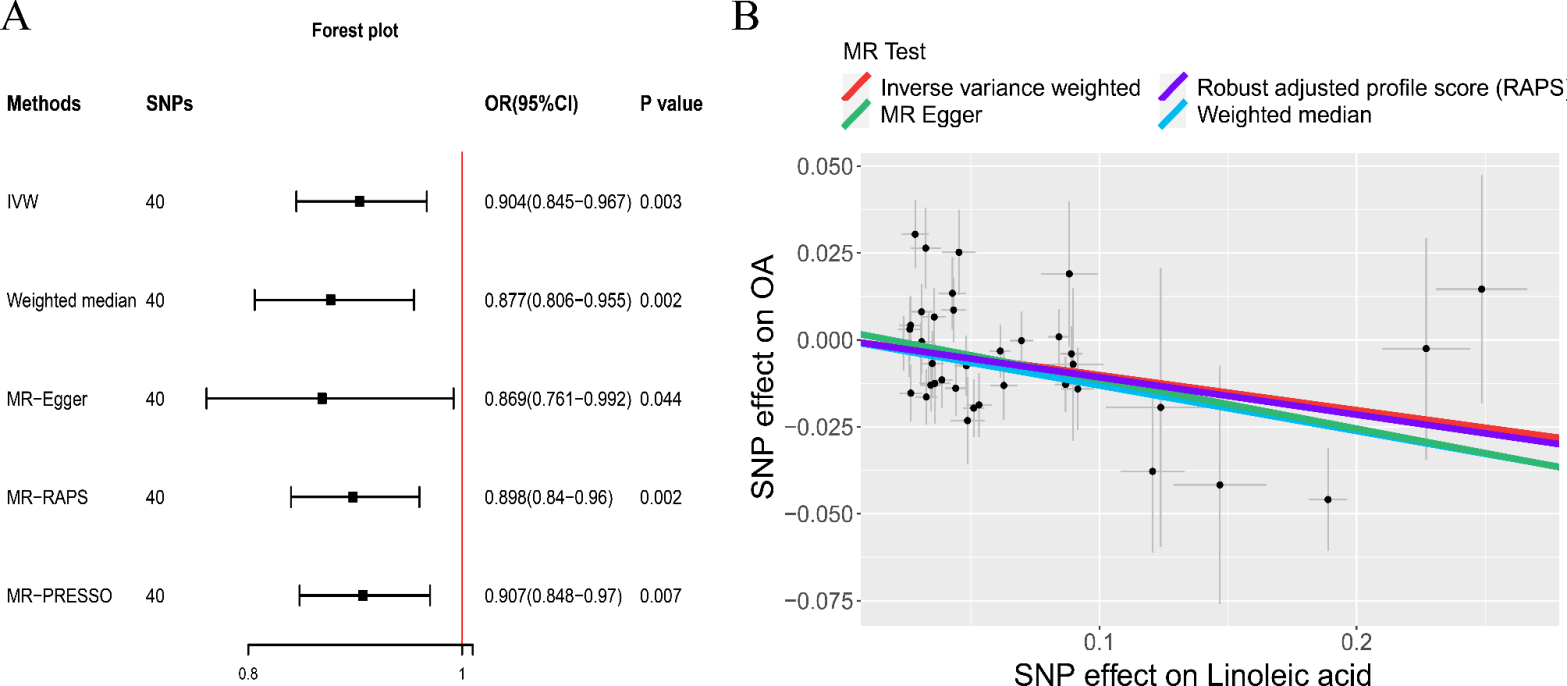
The causal relationship between linoleic acid and osteoarthritis (validation). Forest plot (**a**) Scatter plot (**b**)

Unlike the discovery set, heterogeneity was detected in the validation set (Table 2), so we used the results of the random-effect IVW model. As shown in Fig. 3, the results of the five analysis methods were consistent and statistically significant (IVW: OR = 0.904, 95% CI: 0.845–0.967, *p* = 0.003). The MR-Egger intercept method also showed no horizontal pleiotropy (Table 2). The results of the MR Steiger test and leave-one-out analysis were the same as the discovery set (Supplemental Table S2, Supplemental Figure S2).

**Fig. 4:**
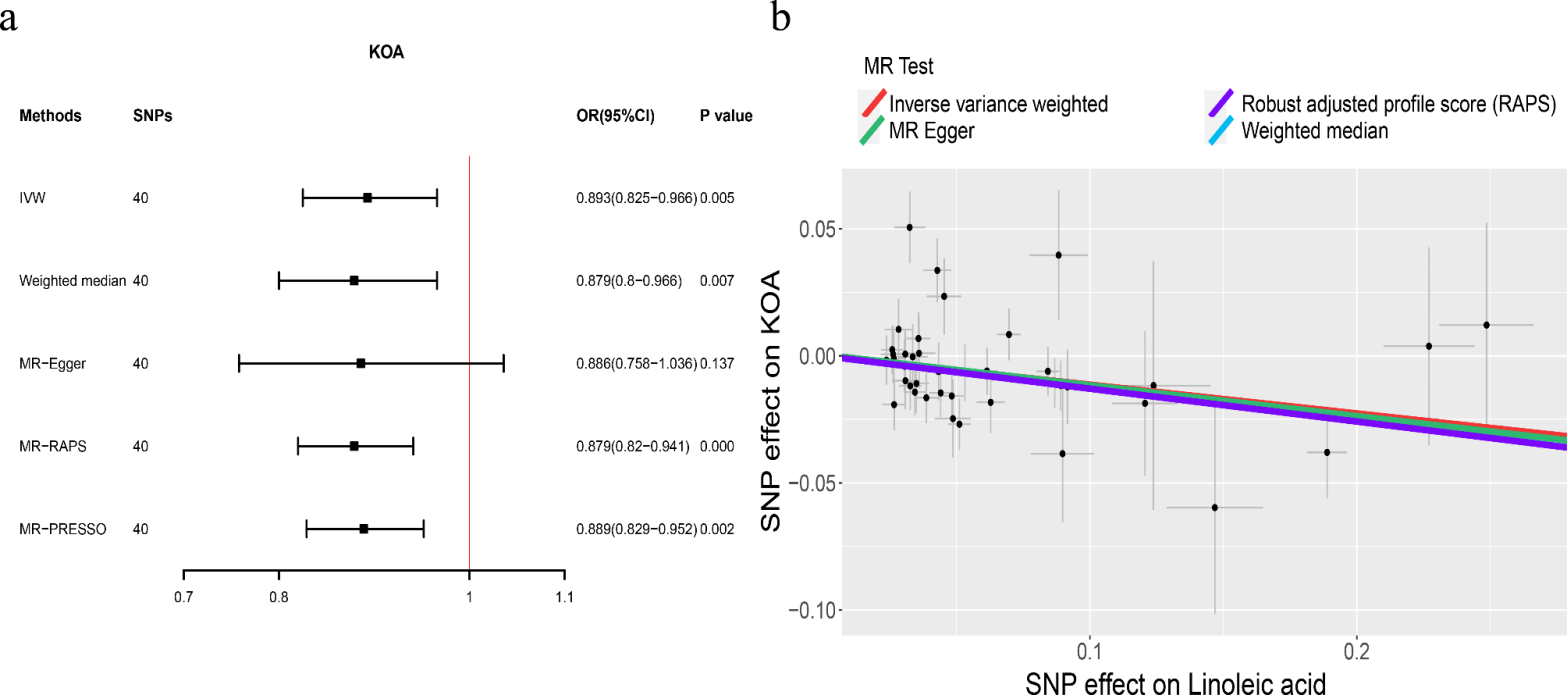
The causal relationship between linoleic acid and KOA. Forest plot (**a**) Scatter plot (**b**)

**Fig. 5:**
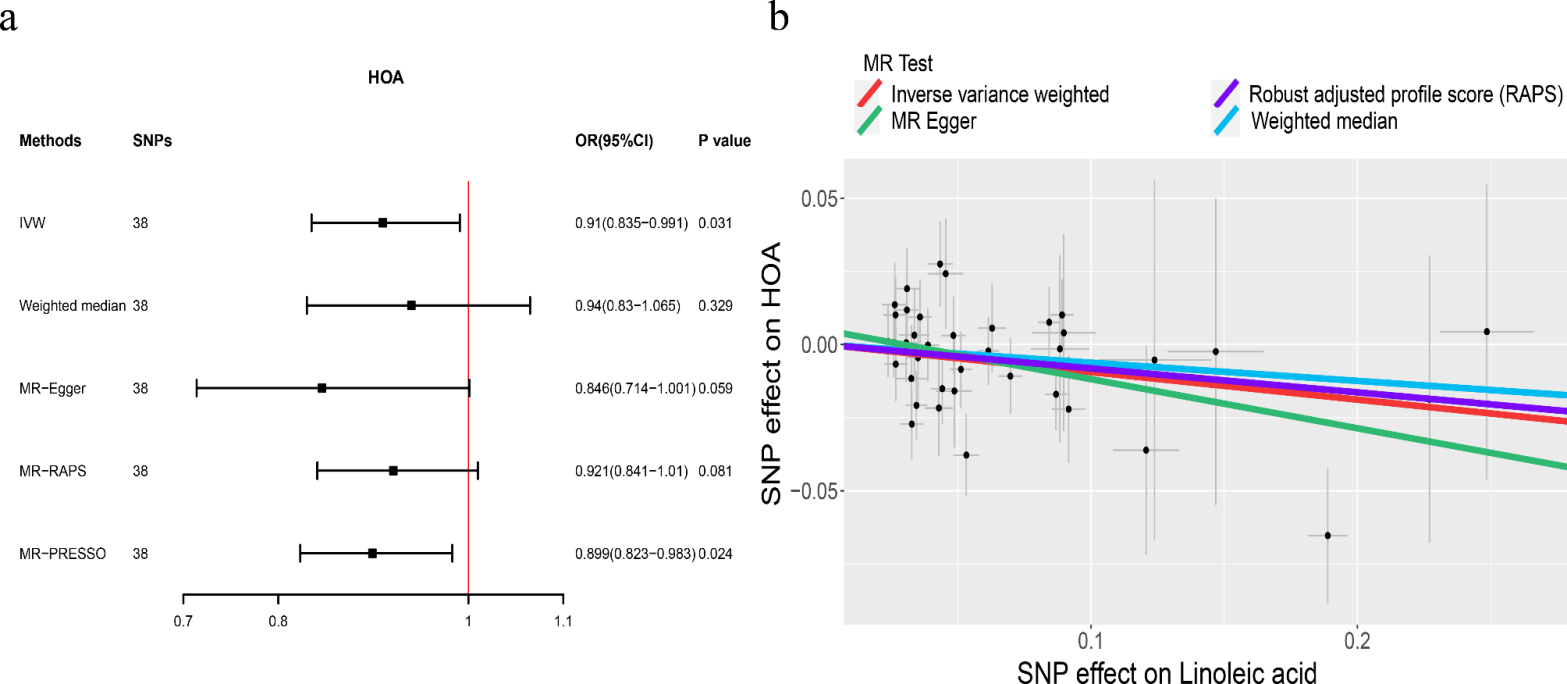
The causal relationship between linoleic acid and HOA. Forest plot (**a**) Scatter plot (**b**)

To further explore the role of LA on different OA subtypes, we selected KOA and HOA for analysis. For KOA, there was no horizontal pleiotropy, although heterogeneity was detected (Table 2). As shown in Fig. 4, the IVW method indicated that genetically predicted higher LA levels were correlated with a significantly lower risk of OA (IVW: OR = 0.893, 95% CI: 0.825–0.966, *p* = 0.005). Similarly, for HOA, the IVW method showed a negative causal relationship with the risk of OA (IVW: OR = 0.91, 95% CI: 0.835–0.991, *p* = 0.031) (Fig.5). Meanwhile, the leave-one-out analysis also showed that single SNP did not lead to causal bias (Supplemental Figures S3, S4).

### GO, KEGG and PPI analysis

**Fig. 6:**
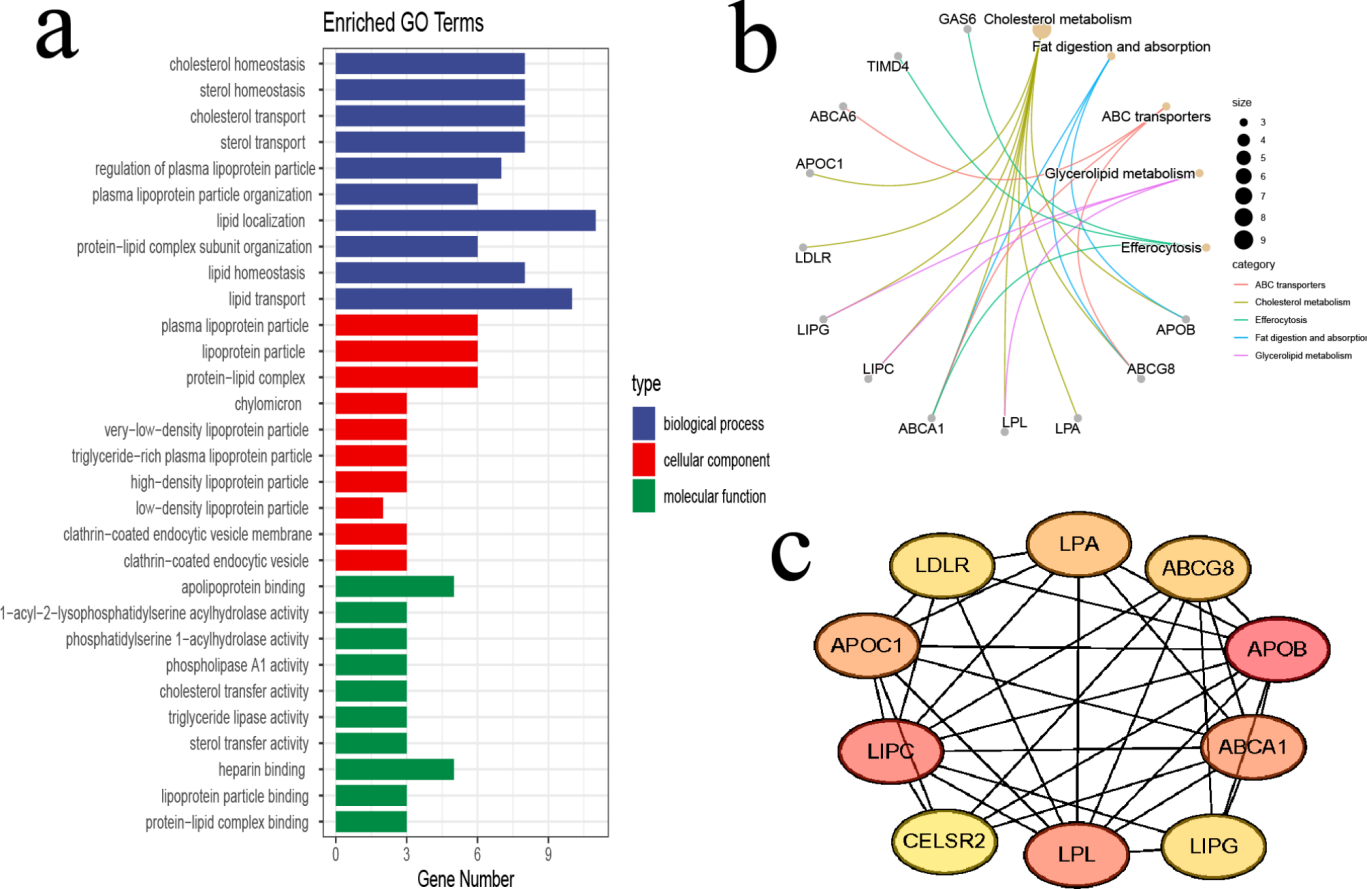
Functional enrichment and PPI of mapping genes. GO enrichment (**a**) KEGG enrichment (**b**) Top 10 genes based on MCC method (**c**)

We organized the genes corresponding to the SNPs screened above according to the information on the Scanner website and the GWAS website, removing some missing values, and obtained a total of 35 mapped genes. The specific gene information is shown in Supplemental Table S3. To further explore the potential biological functions and signaling pathways of mapping genes, we performed GO and KEGG enrichment analysis. The GO analysis results revealed that the mapping genes were mostly enriched in cholesterol homeostasis, sterol homeostasis, cholesterol transport, sterol transport and regulation of plasma lipoprotein particle for biology process (BP); plasma lipoprotein particle, lipoprotein particle, protein-lipid complex, chylomicron and very-low-density lipoprotein particle for cellular component (CC); apolipoprotein binding, 1-acyl-2-lysophosphatidylserine acyl hydrolase activity, phosphatidylserine 1-acyhydrolase activity, phospholipase A1 activity and cholesterol transfer activity for molecular function (MF) (Fig. 6a). Otherwise, KEGG pathway enrichment analysis showed that pathways were mainly associated with cholesterol metabolism, fat digestion and absorption, ABC transporters, glycerolipid metabolism and efferocytosis (Fig. 6b). We then obtained the PPI results from the STRING database and then used the CytoHubba plugin MCC to calculate the score of each node gene. The top 10 genes (APOB, LIPC, LPL, ABCA1, APOC1, LPA, ABCG8, LIPG, LDLR, CELSR2) of the MCC method are shown in Fig. 6c.

## Discussion

In this study, results from both the discovery and validation sets indicated that higher levels of circulating LA were significantly associated with a lower risk of OA. And this finding was further confirmed in the two major OA subtypes (KOA and HOA). Meanwhile, multiple causal analyses and sensitivity analyses further confirmed the reliability of our findings.

LA is the major PUFA found in most Western diets. Since the 1960s, the average intake in Western countries has risen steadily from 2.7 g/day to about 4.9 g/day to 21.0 g/day (about 4–10% of total dietary calories) [30]. The largest increase in intake has been for LA compared to other dietary fatty acids [31]. It is an essential nutrient because it cannot be synthesized in the human body [32]. LA is available from a variety of sources, including many vegetable oils, nuts and seeds, and products made from vegetable oils, such as margarine [33]. Previous studies have concluded that diets high in LA are unhealthy and promote inflammation [34, 35]. There were several possible reasons why LA promotes inflammation: (1) LA itself was metabolized by lipoxygenase to a number of oxidized derivatives, or its various oxidized forms directly stimulated inflammation [36, 37]. (2) promoted the synthesis of arachidonic acid and pro-inflammatory eicosanoids [38, 39]. (3) reduced the conversion of α-linolenic acid to eicosatetraenoic acid (EPA) and/or docosahexaenoic acid (DHA), and reduced the synthesis of anti-inflammatory eicosanoids in EPA and DHA [40]. Fortunately, however, a recently published evidence-based review integrating all available human clinical data showed that there was no significant positive correlation between LA intake and circulating concentrations of several inflammatory markers [41]. In addition, results from a subsequent randomized controlled trial indicated that increased LA intake reduced inflammatory biomarkers in obese participants [42]. This challenged the conventional view that linoleic acid promotes the progression of inflammation.

There are few studies on LA in OA. An in vitro study by Jenniskens et al. showed that LA induced chondrocyte prostaglandin-E2 (PGE2) production in the presence of TNFα, thereby promoting OA progression [7]. A recent study suggested that LA inhibits chondrocyte autophagy, which promotes cartilage degeneration and exacerbates OA progression [8]. In contrast, a recent cohort study found that LA levels were negatively associated with hand OA severity [11]. Another exploratory metabolomic study showed that LA levels were instead elevated after OA was inhibited [10]. These supported our conclusion. Interestingly, conjugated linoleic acid (CLA), a group of positional and geometric (cis or trans) isomers of LA with conjugated double bonds, has been shown to exert anti-inflammatory effects in OA [43, 44]. It is noteworthy that OA has not been specifically typed in this literature. It is well known that OA is a type of arthritis that leads to joint pain and dysfunction, and pathological changes often involve destruction of cartilage, remodeling of subchondral bone, and joint inflammation [1]. OA can involve all joints throughout the body, with the knee and hip joints being the most common. Our study not only found a causal relationship between LA and OA overall, but also analyzed the two most common subtypes, KOA and HOA, to further confirm this causal relationship and make the results more reliable.

It is well known that LA is one of the major members of omega-6 PUFA. Regarding omega-6 PUFA, many studies have demonstrated its pro-inflammatory role in OA. In mice with surgically induced OA, omega-6 PUFA independently increased the severity of osteoarthritis, heterotopic ossification, and scar tissue formation [45]. In a Multicenter Osteoarthritis Study (MOST), Baker et al. found that omega-6 was positively associated with knee synovitis [46]. A lower dietary ratio of omega-6 to omega-3 FA has been shown to inhibit MMP-13 in inflammatory arthritis in rats [47]. Fortunately, a recent study measuring the fatty acid composition of knee synovial fluid showed a trend toward decreased omega-6 in the synovial fluid of OA patients compared to healthy individuals, which also seems to provide limited support for our conclusions [48].

To further explore the potential biological mechanisms and pathways by which LA affects OA, we performed GO and KEGG enrichment analysis on the genes mapped by SNPs. The GO and KEGG results showed that these genes were mainly related to the metabolism and homeostasis of cholesterol, lipids, and lipoproteins, etc. KEGG also suggested that efferocytosis might also be involved in the regulation of OA by LA. We next screened to obtain the TOP10 hub genes. Although some previous studies have shown that APOB is not or positively associated with OA [49, 50], a recently published MR study found that APOB has a genetically protective effect on OA [51], which is consistent with our results. However, the mechanism of this protective effect is not clear and further studies are needed. LIPC may influence the development of OA by participating in bone metabolism and calcification [52]. Wang et al. found that overexpression of ABCA1 enhanced OA chondrocyte activity and alleviated inflammation [53]. Peng et al. found that the SIRT1/FOXO3a/ABCA1 axis improved OA by ameliorating cholesterol metabolism dysregulation [54]. LPA may improve OA progression by stabilizing YAP protein and inhibiting the Hippo signaling pathway [55, 56]. These studies provide a basis for future exploration of the molecular mechanisms by which LA regulates OA.

The main advantage of this study is that the use of the MR method reduces and avoids confounding and reverse causality, which are common in traditional observational studies. The participants in this study were of European ancestry, thus reducing the bias of racial differences. This study included both the discovery and validation sets, making the results more reliable. In addition, the use of multiple MR analysis methods and sensitivity analysis improved the credibility of our results.

However, this study also has several limitations. First, this study was limited to European ancestry, so other races may not be applicable. Second, the publicly available data used in this study did not include some specific factors, such as age and sex, so we could not perform subgroup analysis. Finally, there was heterogeneity among the IVs used in the validation set; however, no horizontal pleiotropy was detected in the MR-Egger test, which is unlikely to bias the results.

## Conclusion

In conclusion, our study suggests that higher levels of circulating LA are associated with a lower risk of OA, KOA and HOA by MR analysis. Validation and sensitivity analyses show that our conclusions are very robust. These findings may provide new ideas for the treatment of OA, but further studies are needed to investigate the potential mechanisms between LA and OA.

## Electronic supplementary material

Below is the link to the electronic supplementary material.

Supplementary Material 1

## Data Availability

The data used in this study are publicly available (https://gwas.mrcieu.ac.uk/) or can be obtained from the corresponding authors upon reasonable request.
