# Supplementary material for: Genetically predicted circulating linoleic acid levels and risk of osteoarthritis: a two-sample mendelian randomization study
Source: BMC Musculoskelet Disord. 2024 Nov 13;25:903. doi: 10.1186/s12891-024-08018-4 (PMC11577923; doi:10.1186/s12891-024-08018-4)
Supplement: Supplementary file 1 — Supplementary Material 1 [file 12891_2024_8018_MOESM1_ESM.pdf]

Supplemental Table S1. Single nucleotide polymorphisms (SNP) associated with linoleic acid

SNP	Exposure	Effect allele	Other allele	EAF	Beta	SE	P value	Sample size	R2	F statistic
rs34232196	Linoleic acid	T	C	0.245325	-0.0305851	0.00474221	8.30E-11	114999	0.000361582	41.59663943
rs199900492	Linoleic acid	A	C	0.484009	-0.0291075	0.00406151	4.60E-14	114999	0.000446423	51.36115228
rs534417	Linoleic acid	G	A	0.875005	0.0359986	0.00610468	2.50E-09	114999	0.000302287	34.77326058
rs2986164	Linoleic acid	A	G	0.535856	-0.0266721	0.00442883	5.00E-10	114999	0.000315287	36.26906844
rs1002687	Linoleic acid	A	G	0.644748	0.0868452	0.0042312	1.80E-97	114999	0.003649912	421.2738787
rs602633	Linoleic acid	G	T	0.78295	0.0531806	0.00490259	7.50E-28	114999	0.001022156	117.667172
rs35633876	Linoleic acid	T	G	0.481841	-0.0344611	0.00406887	2.40E-17	114999	0.00062337	71.73162068
rs4665972	Linoleic acid	C	T	0.604642	-0.0513405	0.00416674	1.30E-35	114999	0.00131844	151.8194397
rs693	Linoleic acid	A	G	0.520883	0.0614441	0.00405281	3.40E-54	114999	0.001994742	229.851788
rs4299376	Linoleic acid	T	G	0.676279	-0.0387395	0.00433864	2.50E-19	114999	0.000692797	79.72614963
rs2389599	Linoleic acid	C	G	0.524817	0.0230636	0.00407881	6.80E-09	114999	0.000277954	31.9732839
rs9848779	Linoleic acid	G	C	0.533474	-0.0214701	0.00408376	3.40E-08	114999	0.000240298	27.64061568
rs13108218	Linoleic acid	G	A	0.615417	-0.0327027	0.00419342	5.90E-16	114999	0.000528575	60.81777063
rs7707394	Linoleic acid	A	G	0.357106	0.0316688	0.00422065	3.90E-14	114999	0.000489326	56.29949971
rs4704210	Linoleic acid	C	G	0.374212	0.0511244	0.00418591	2.00E-35	114999	0.001295447	149.1683464
rs6882345	Linoleic acid	A	G	0.632863	0.0441266	0.00419712	2.00E-26	114999	0.000960256	110.5345784
rs35599691	Linoleic acid	A	G	0.577598	0.0350233	0.00475488	6.60E-12	114999	0.000471559	54.25440517
rs13217434	Linoleic acid	C	G	0.259322	0.0433091	0.00471025	4.30E-20	114999	0.000734611	84.54158799
rs3011437	Linoleic acid	G	T	0.294371	0.0357926	0.0044745	1.30E-16	114999	0.000556111	63.98784279
rs186696265	Linoleic acid	T	C	0.014358	-0.227024	0.0170817	2.20E-41	114999	0.001533631	176.6368707
rs4947302	Linoleic acid	T	C	0.064792	0.0459621	0.00824431	4.80E-10	114999	0.000270196	31.08072204

rs36018387	Linoleic acid	T	C	0.105092	-0.0442699	0.00662522	6.40E-12	114999	0.000388109	44.64948701
rs7750288	Linoleic acid	G	A	0.285011	0.0265974	0.0044814	1.10E-09	114999	0.000306213	35.22499578
rs55747707	Linoleic acid	A	G	0.203615	-0.0449899	0.00504536	1.50E-18	114999	0.000690958	79.51439084
rs1461729	Linoleic acid	G	A	0.899221	0.0703705	0.00673534	5.40E-26	114999	0.000948323	109.159673
rs112875651	Linoleic acid	A	G	0.392346	-0.0510959	0.00420654	5.00E-35	114999	0.00128136	147.5441723
rs7816447	Linoleic acid	C	T	0.100848	-0.0487086	0.00671655	8.20E-13	114999	0.000457116	52.5918914
rs6471717	Linoleic acid	A	G	0.663091	-0.0308556	0.00429616	1.40E-13	114999	0.000448351	51.58305948
rs115478735	Linoleic acid	T	A	0.183315	0.0428585	0.00523882	1.20E-17	114999	0.000581648	66.92786645
rs11789603	Linoleic acid	T	C	0.108831	0.0454099	0.00651811	1.70E-12	114999	0.000421872	48.53530021
rs4008004	Linoleic acid	A	C	0.221842	0.0309046	0.00489606	1.90E-10	114999	0.000346344	39.84306969
rs2740488	Linoleic acid	C	A	0.265321	-0.0482851	0.00459865	1.00E-25	114999	0.000957757	110.2466893
rs148063610	Linoleic acid	C	T	0.763414	-0.0313506	0.00486658	9.70E-11	114999	0.000360739	41.49960661
rs10838724	Linoleic acid	T	G	0.368821	-0.0262357	0.00424528	1.60E-10	114999	0.000331997	38.19202035
rs174564	Linoleic acid	G	A	0.347013	0.0842755	0.00424412	5.10E-88	114999	0.003417014	394.3005347
rs200671503	Linoleic acid	T	A	0.941966	0.053839	0.00870398	2.70E-10	114999	0.000332598	38.26116598
rs964184	Linoleic acid	C	G	0.867229	-0.145606	0.00596821	2.00E-136	114999	0.005149135	595.2101804
rs141469619	Linoleic acid	G	A	0.01014	0.123842	0.0213968	3.80E-10	114999	0.000291218	33.49949496
rs4766578	Linoleic acid	A	T	0.503267	0.0255029	0.00405844	1.90E-10	114999	0.000343256	39.48761252
rs7139079	Linoleic acid	A	G	0.592501	-0.0259545	0.00413291	4.80E-10	114999	0.000342823	39.43786878
rs6602911	Linoleic acid	T	C	0.360079	0.0238048	0.00421825	1.40E-08	114999	0.000276854	31.84670051
rs261290	Linoleic acid	C	T	0.654653	-0.089118	0.00426927	7.90E-99	114999	0.003774741	435.7361779
rs633695	Linoleic acid	G	A	0.292348	0.0697	0.00446965	2.00E-54	114999	0.00211012	243.1747751
rs247617	Linoleic acid	A	C	0.323625	0.0506744	0.00433307	6.50E-33	114999	0.001187888	136.768417
rs9302635	Linoleic acid	C	T	0.182464	-0.0283511	0.00523184	3.40E-08	114999	0.000255285	29.36506184
rs12948283	Linoleic acid	C	G	0.297323	0.0263025	0.00465578	6.40E-09	114999	0.000277456	31.91605155
rs740516	Linoleic acid	G	C	0.151132	-0.03364	0.00568287	1.90E-09	114999	0.000304614	35.04100323

rs77960347	Linoleic acid	G	A	0.013239	0.248604	0.0176915	1.10E-45	114999	0.001714146	197.4635364
rs9304381	Linoleic acid	T	C	0.818434	0.0628471	0.00525856	1.00E-33	114999	0.00124052	142.8357166
rs79429216	Linoleic acid	A	G	0.012713	0.146817	0.0180405	1.60E-16	114999	0.000575588	66.23012141
rs142158911	Linoleic acid	A	G	0.116705	-0.0915875	0.00634525	1.20E-48	114999	0.001808401	208.3410908
rs56322906	Linoleic acid	A	G	0.035157	-0.0883014	0.0110068	2.90E-15	114999	0.00055934	64.35955435
rs58542926	Linoleic acid	T	C	0.074383	-0.109822	0.00772171	7.50E-48	114999	0.001755878	202.2794308
rs1065853	Linoleic acid	T	G	0.080578	-0.188865	0.00746999	1.90E-143	114999	0.005527916	639.238502
rs1081105	Linoleic acid	C	A	0.027624	0.120669	0.0123858	8.20E-23	114999	0.00082469	94.91684664
rs2378390	Linoleic acid	A	G	0.140871	-0.0324917	0.00584433	7.00E-09	114999	0.000268698	30.90832062
rs1883711	Linoleic acid	C	G	0.031191	0.089775	0.0119031	3.70E-15	114999	0.000494404	56.88407013

Note: LA, Linoleic acid; SNP, single nucleotide polymorphism; EAF, effect allele frequency; MAF, minor allele frequency; SE, standard error.

Supplemental Table S2. Results of MR-Steiger infiltering test.

SNP	Exposure	Outcome	Steiger_dir	Steiger_pval
rs1002687	Linoleic acid	OA(discovery)	TRUE	1.13E-69
rs1081105	Linoleic acid	OA(discovery)	TRUE	3.60E-16
rs11789603	Linoleic acid	OA(discovery)	TRUE	6.41E-08
rs13108218	Linoleic acid	OA(discovery)	TRUE	1.07E-10
rs141469619	Linoleic acid	OA(discovery)	TRUE	2.26E-05
rs142158911	Linoleic acid	OA(discovery)	TRUE	1.16E-37
rs174564	Linoleic acid	OA(discovery)	TRUE	1.68E-67
rs186696265	Linoleic acid	OA(discovery)	TRUE	7.20E-31
rs1883711	Linoleic acid	OA(discovery)	TRUE	4.32E-11
rs2389599	Linoleic acid	OA(discovery)	TRUE	1.93E-05
rs261290	Linoleic acid	OA(discovery)	TRUE	1.30E-74

rs2740488	Linoleic acid	OA(discovery)	TRUE	2.93E-20
rs2986164	Linoleic acid	OA(discovery)	TRUE	2.68E-07
rs3011437	Linoleic acid	OA(discovery)	TRUE	3.93E-12
rs34232196	Linoleic acid	OA(discovery)	TRUE	8.09E-08
rs35633876	Linoleic acid	OA(discovery)	TRUE	2.86E-10
rs4008004	Linoleic acid	OA(discovery)	TRUE	7.10E-08
rs4299376	Linoleic acid	OA(discovery)	TRUE	1.78E-14
rs534417	Linoleic acid	OA(discovery)	TRUE	1.90E-06
rs56322906	Linoleic acid	OA(discovery)	TRUE	1.91E-12
rs602633	Linoleic acid	OA(discovery)	TRUE	2.47E-18
rs633695	Linoleic acid	OA(discovery)	TRUE	1.42E-43
rs6471717	Linoleic acid	OA(discovery)	TRUE	4.47E-09
rs6602911	Linoleic acid	OA(discovery)	TRUE	3.39E-06
rs6882345	Linoleic acid	OA(discovery)	TRUE	1.52E-20
rs693	Linoleic acid	OA(discovery)	TRUE	1.98E-38
rs7139079	Linoleic acid	OA(discovery)	TRUE	3.33E-06
rs740516	Linoleic acid	OA(discovery)	TRUE	9.04E-07
rs7750288	Linoleic acid	OA(discovery)	TRUE	3.89E-07
rs77960347	Linoleic acid	OA(discovery)	TRUE	1.81E-32
rs7816447	Linoleic acid	OA(discovery)	TRUE	6.28E-10
rs79429216	Linoleic acid	OA(discovery)	TRUE	3.75E-12
rs9302635	Linoleic acid	OA(discovery)	TRUE	4.81E-05
rs9304381	Linoleic acid	OA(discovery)	TRUE	1.71E-22
rs9848779	Linoleic acid	OA(discovery)	TRUE	0.000131208
rs1002687	Linoleic acid	OA(validation)	TRUE	6.87E-68
rs1065853	Linoleic acid	OA(validation)	TRUE	3.11E-97

rs1081105	Linoleic acid	OA(validation)	TRUE	3.50E-15
rs115478735	Linoleic acid	OA(validation)	TRUE	3.04E-11
rs11789603	Linoleic acid	OA(validation)	TRUE	1.82E-07
rs12948283	Linoleic acid	OA(validation)	TRUE	1.26E-06
rs13108218	Linoleic acid	OA(validation)	TRUE	2.85E-09
rs13217434	Linoleic acid	OA(validation)	TRUE	1.25E-14
rs141469619	Linoleic acid	OA(validation)	TRUE	9.58E-07
rs142158911	Linoleic acid	OA(validation)	TRUE	2.24E-34
rs174564	Linoleic acid	OA(validation)	TRUE	9.97E-69
rs186696265	Linoleic acid	OA(validation)	TRUE	9.05E-32
rs1883711	Linoleic acid	OA(validation)	TRUE	6.58E-11
rs2378390	Linoleic acid	OA(validation)	TRUE	0.000111042
rs2389599	Linoleic acid	OA(validation)	TRUE	2.04E-05
rs261290	Linoleic acid	OA(validation)	TRUE	2.49E-74
rs2740488	Linoleic acid	OA(validation)	TRUE	5.79E-19
rs2986164	Linoleic acid	OA(validation)	TRUE	7.99E-06
rs3011437	Linoleic acid	OA(validation)	TRUE	1.85E-11
rs34232196	Linoleic acid	OA(validation)	TRUE	3.76E-08
rs35599691	Linoleic acid	OA(validation)	TRUE	6.19E-10
rs35633876	Linoleic acid	OA(validation)	TRUE	2.03E-11
rs4008004	Linoleic acid	OA(validation)	TRUE	2.64E-08
rs4299376	Linoleic acid	OA(validation)	TRUE	4.30E-13
rs4704210	Linoleic acid	OA(validation)	TRUE	2.60E-22
rs534417	Linoleic acid	OA(validation)	TRUE	2.40E-06
rs56322906	Linoleic acid	OA(validation)	TRUE	2.38E-11
rs602633	Linoleic acid	OA(validation)	TRUE	4.76E-18

rs633695	Linoleic acid	OA(validation)	TRUE	2.84E-43
rs6471717	Linoleic acid	OA(validation)	TRUE	3.89E-09
rs6602911	Linoleic acid	OA(validation)	TRUE	7.89E-07
rs6882345	Linoleic acid	OA(validation)	TRUE	2.06E-17
rs693	Linoleic acid	OA(validation)	TRUE	6.33E-40
rs7139079	Linoleic acid	OA(validation)	TRUE	7.14E-08
rs740516	Linoleic acid	OA(validation)	TRUE	2.79E-07
rs7750288	Linoleic acid	OA(validation)	TRUE	5.09E-07
rs77960347	Linoleic acid	OA(validation)	TRUE	2.07E-34
rs7816447	Linoleic acid	OA(validation)	TRUE	2.61E-08
rs79429216	Linoleic acid	OA(validation)	TRUE	3.12E-11
rs9302635	Linoleic acid	OA(validation)	TRUE	0.000748118
rs9304381	Linoleic acid	OA(validation)	TRUE	2.15E-23
rs9848779	Linoleic acid	OA(validation)	TRUE	2.24E-05

Supplemental Table S3. Genes mapped by SNPs

snp	symbol
rs34232196	BSND
rs534417	ASAP3
rs2986164	RHD
rs1002687	DOCK7
rs602633	CELSR2
rs35633876	RPS16P2
rs693	APOB

rs4299376	ABCG8
rs2389599	DHRS9
rs9848779	PAQR9
rs13108218	HGFAC
rs6882345	TIMD4
rs3011437	LPA
rs186696265	PLG
rs7750288	IGF2R
rs7816447	LPL
rs6471717	UBXN2B
rs11789603	ABCA1
rs4008004	TTC39B
rs2740488	ABCA1
rs174564	FADS2
rs141469619	SIK3
rs7139079	HNF1A
rs6602911	GAS6
rs261290	ALDH1A2
rs633695	LIPC
rs9302635	DHX38
rs740516	ABCA6
rs77960347	LIPG
rs9304381	SMUG1P1
rs79429216	APOC4-APOC2
rs142158911	LDLR
rs56322906	DOCK6

rs1081105	APOC1
rs1883711	LINC01728

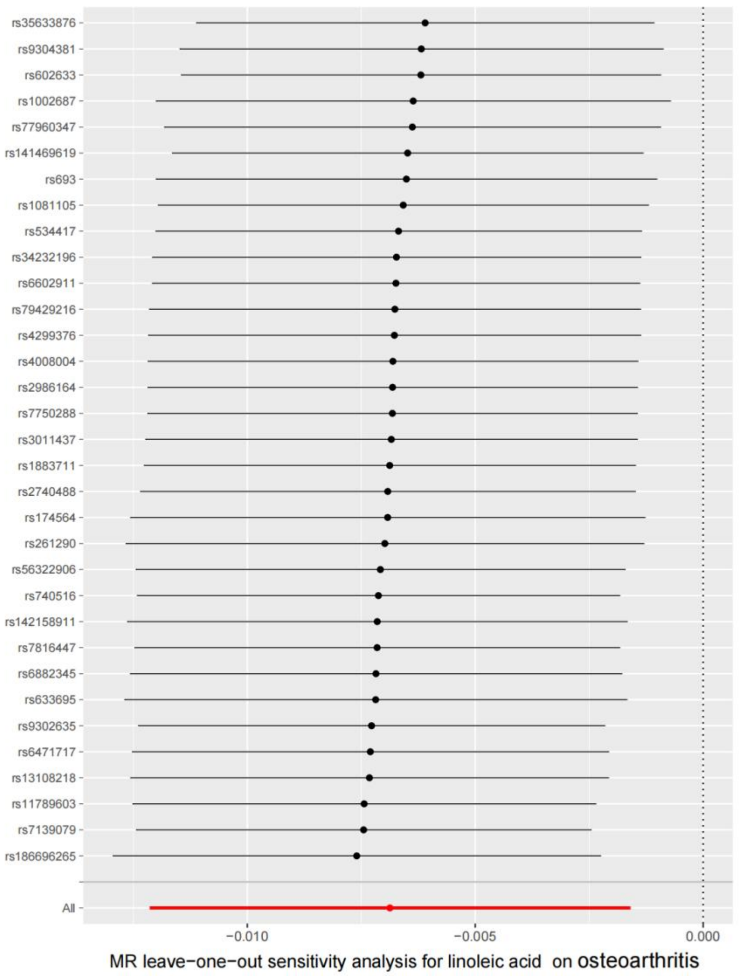

Fig. S1 Leave-one-out analysis of linoleic acid on osteoarthritis (discovery).

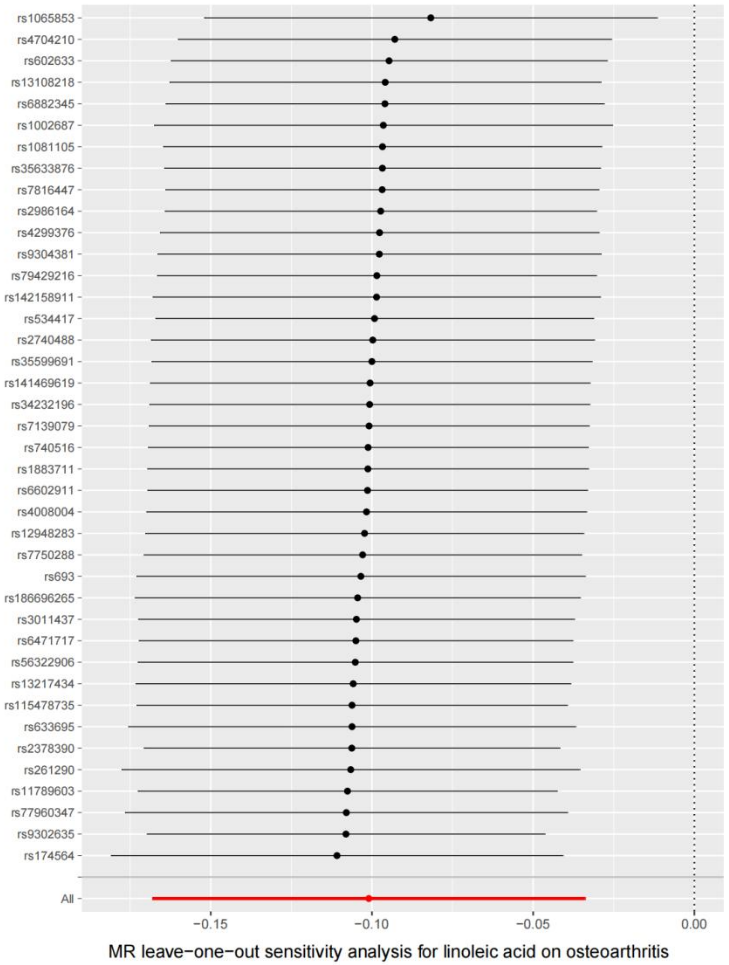

Fig. S2 Leave-one-out analysis of linoleic acid on osteoarthritis (validation).

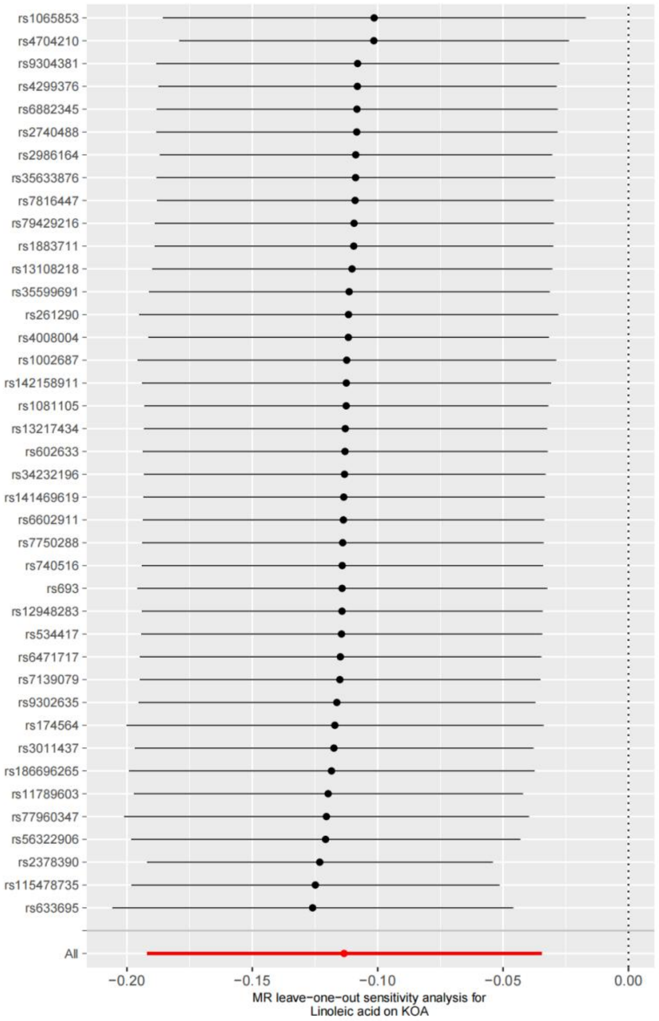

Fig. S3 Leave-one-out analysis of linoleic acid on KOA.

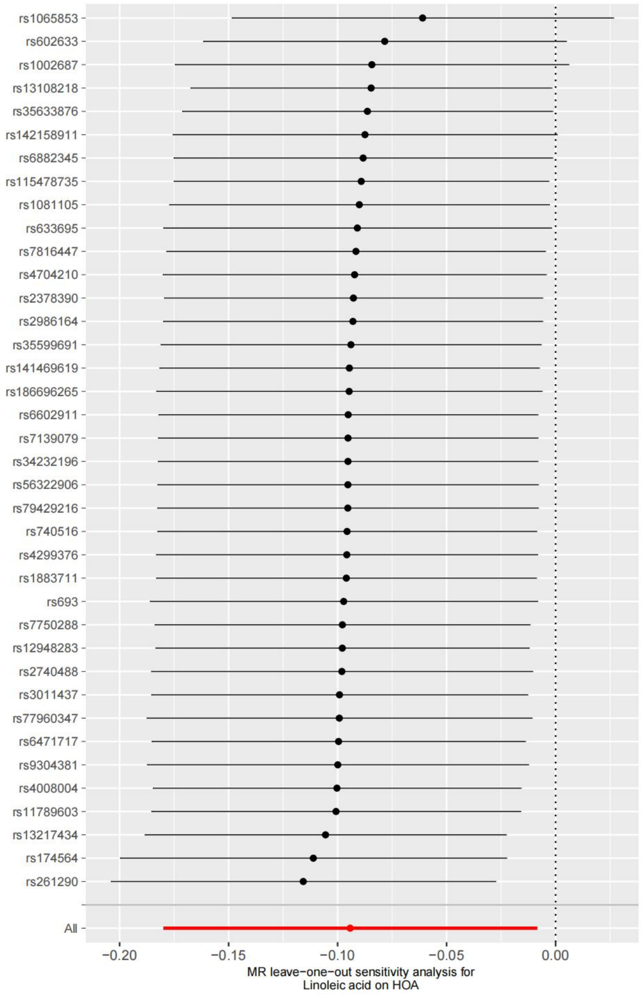

Fig. S4 Leave-one-out analysis of linoleic acid on HOA.
